# Exon-skipping and mRNA decay in human liver tissue: molecular consequences of pathogenic bile salt export pump mutations

**DOI:** 10.1038/srep24827

**Published:** 2016-04-26

**Authors:** Carola Dröge, Heiner Schaal, Guido Engelmann, Daniel Wenning, Dieter Häussinger, Ralf Kubitz

**Affiliations:** 1 Department of Gastroenterology, Hepatology and Infectious Diseases, University Hospital, Heinrich Heine University, Düsseldorf, Germany; 2 Institute of Virology, Heinrich Heine University, Düsseldorf, Germany; 3 Department of Pediatrics, Lukashospital, Neuss, Germany; 4 Department of General Pediatrics, University Hospital, Heidelberg, Germany; 5 Medical Clinic I, Bethanien Hospital, Moers, Germany

## Abstract

The bile salt export pump BSEP mediates bile formation. Over 150 BSEP mutations are associated with progressive familial intrahepatic cholestasis type 2 (PFIC-2), with few characterised specifically. We examined liver tissues from two PFIC-2 patients compound heterozygous for the splice-site mutation c.150 + 3A > C and either c.2783_2787dup5 resulting in a frameshift with a premature termination codon (child 1) or p.R832C (child 2). Splicing was analysed with a minigene system and mRNA sequencing from patients’ livers. Protein expression was shown by immunofluorescence. Using the minigene, c.150 + 3A > C causes complete skipping of exon 3. In liver tissue of child 1, c.2783_2787dup5 was found on DNA but not on mRNA level, implying nonsense-mediated mRNA decay (NMD) when c.2783_2787dup5 is present. Still, BSEP protein as well as mRNA with and without exon 3 were detectable and can be assigned to the c.150 + 3A > C allele. Correctly spliced transcripts despite c.150 + 3A > C were also confirmed in liver of child 2. In conclusion, we provide evidence (1) for effective NMD due to a BSEP frameshift mutation and (2) partial exon-skipping due to c.150 + 3A > C. The results illustrate that the extent of exon-skipping depends on the genomic and cellular context and that regulation of splicing may have therapeutic potential.

The bile salt export pump BSEP (gene symbol: *ABCB11*) belongs to subfamily B (MDR/TAP) of adenosine triphosphate-binding cassette (ABC) transporters. BSEP is exclusively localised at the canalicular membrane of hepatocytes and mediates bile salt-dependent bile flow by transporting bile salts from the hepatocyte into the bile canaliculus, where bile salts form mixed micelles together with cholesterol and phospholipids^1^. Human BSEP is composed of 1321 amino acids encoded by the *ABCB11* gene, which is located on chromosome 2 (2q24)^2^ and consists of a leading non-coding and 27 coding exons. BSEP mutations are the basis of cholestatic liver diseases of varying severity ranging from milder forms as intrahepatic cholestasis of pregnancy (ICP)^3^,^4^,^5^ or benign recurrent intrahepatic cholestasis type 2 (BRIC-2)^6^,^7^ to progressive familial intrahepatic cholestasis type 2 (PFIC-2) often resulting in end-stage liver disease in early childhood necessitating liver transplantation^2^,^8^,^9^,^10^,^11^. More than 290 genetic variants of BSEP are known and about 150 mutations were identified to be associated with PFIC-2 including missense mutations, deletions, insertions, frameshift and nonsense mutations with premature termination codons (PTCs) as well as splice-site mutations (for review see^12^). Donor and acceptor splice-sites specify exon boundaries, which are not only defined by the GT-AG rule^13^, with GT at the 5′ end and AG at the 3′ end of an intron, but also by consensus sequences where the first six intronic positions of the donor splice-site are mostly GT(A/G)AGT^14^. Most influencing splice-site mutations concern +1/+2 (GT) or −1/−2 (AG) intron positions^15^. Nevertheless, more distal nucleotide exchanges within the consensus sequences are also known to be relevant. The putative splice-site mutation c.150 + 3A > C (“c.” for “coding DNA”) shown in two patients with a PFIC-2 phenotype leads to an exchange of adenine to cytosine at the third intron position downstream to *ABCB11* coding exon 3. In the present study, the impact of this more distally located *ABCB11* donor splice-site mutation was proven not only in cultured cells by a minigene splicing assay but also by messenger RNA (mRNA) analyses from liver tissue of two patients with PFIC-2 phenotypes.

## Results

### Genetic analysis

**Figure 1:**
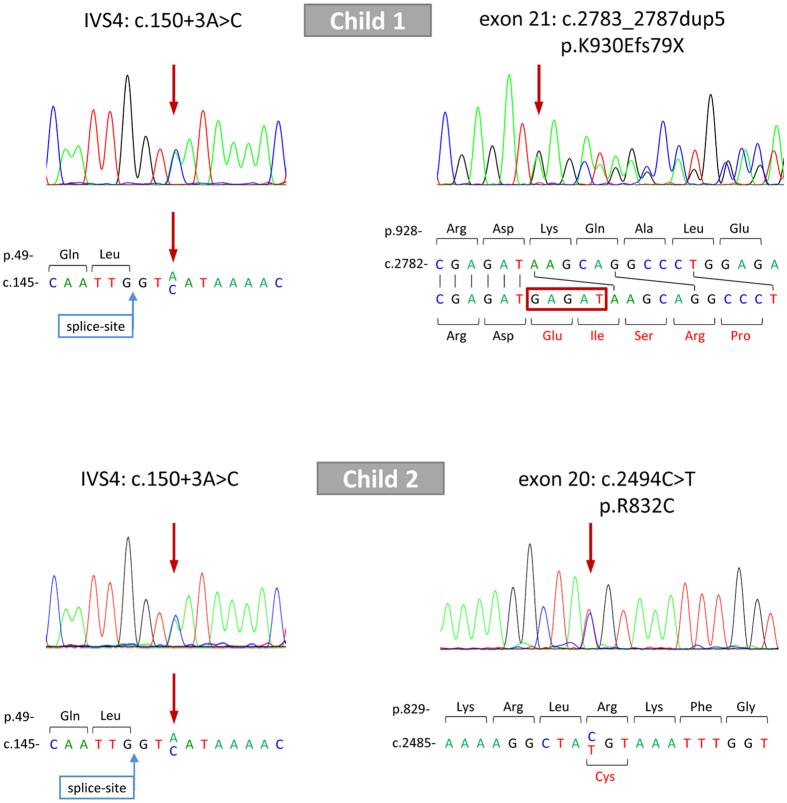
BSEP (*ABCB11*) mutations of two children with PFIC-2. Sequencing of all 27 coding exons with adjacent intron regions of *ABCB11* from gDNA revealed two relevant variants for child 1 (upper panel) and child 2 (lower panel) compared to reference sequence NM_003742.2 (Gene ID: 8647, first translated exon denoted as exon 1, adenine from ATG counted as c.1). Both children were compound heterozygous for the donor splice-site mutation c.150 + 3A > C as well as one exonic mutation. Child 1 had a duplication of GAGAT in exon 21 (c.2783_2787dup5) resulting in a frameshift with a premature termination codon (p.K930fs79X). Child 2 had a nucleotide exchange in exon 20 (c.2494C > T) leading to the missense mutation p.R832C. Corresponding sequences on nucleotide (c.) and protein (p.) level are shown in detail below. IVS4: intervening sequence, intron surrounded by coding exons 3 and 4.

Male child 1 was liver transplanted at the age of 3 due to progressive cholestatic liver disease with normal gamma-glutamyltransferase (gGT) levels. BSEP disease/PFIC-2 was considered, and sequencing of *ABCB11* revealed a heterozygous duplication of five nucleotides (GAGAT) in coding exon 21 (c.2783_2787dup5) (Fig. 1). This mutation, inherited by the father, causes a frameshift and a premature termination codon (PTC) after 78 altered codons (p.K930Efs79X; “p.” for “protein sequence”). Furthermore, the heterozygous intronic mutation c.150 + 3A > C (Fig. 1; transmitted by the mother) concerning the donor splice-site of coding exon 3 was found in addition to two frequent polymorphisms (p.V444A and p.A1028A) and five more intronic variants further away from the splice-sites. All detected BSEP variants of both children are listed in Supplementary Table S1.

Child 2 (male) presented with pruritus, fatigue and elevated bile salt concentrations at 2.5 years of age. gGT levels were always within normal ranges, therefore low gGT-PFIC was assumed. *ABCB11* sequencing showed a heterozygous exchange of cytosine to thymine at position 2494 in coding exon 20 (c.2494C > T) leading to a missense mutation with arginine replaced by cysteine at amino acid position 832 (p.R832C; Fig. 1). This mutation was inherited by the mother whereas the splice-site mutation c.150 + 3A > C was inherited by the father (Fig. 1). Additionally, p.V444A and p.A1028A as well as four other intronic variants in a distance ≥15 nucleotides from the splice-sites were detected (Supplementary Table S1). Child 2 was listed for liver transplantation but was removed from the list two years later because cholestasis was effectively controlled by budesonide^16^.

### *In silico* analysis

The intrinsic strength of the exon 3 splice donor and its mutation c.150 + 3A > C was calculated *in silico*. Wildtype sequence analysis by Berkeley Drosophila Genome Project (http://www.fruitfly.org/seq_tools/splice.html^17^) resulted in 0.41 (possible values: 0 to 1) indicating a weak donor splice-site *per se*. It was no longer recognised in presence of c.150 + 3A > C, suggesting that this mutation leads to a disruption of this splice-site. In line with this, the cytosine at the third intron position was predicted to cause aberrant splicing with high probability according to HBond score algorithm (http://uni-duesseldorf.de/rna/index.php)^18^,^19^.

### Splicing analysis by a minigene assay

**Figure 2:**
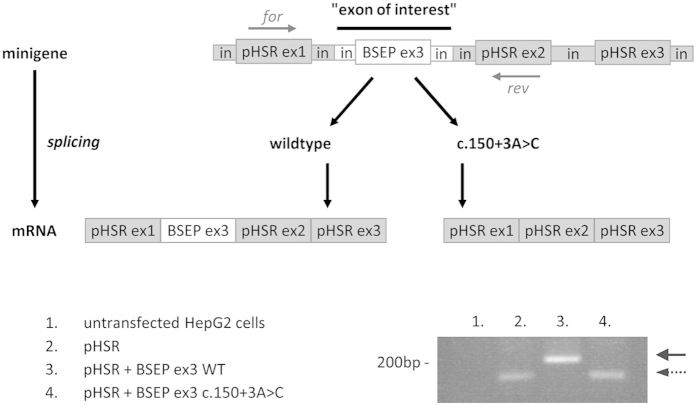
Analysis of c.150 + 3A > C by a minigene splicing assay. HepG2 cells were transiently transfected with a minigene plasmid (pHSR) and pHSR containing BSEP exon 3 with flanking intron regions, wildtype (WT) and mutant (c.150 + 3A > C), respectively. After transfection, RNA preparation, reverse transcription and PCR, agarose gel electrophoresis was performed. Lower bands (dashed arrow) include parts of the spliced pHSR exons 1 and 2 (165 bp) whereas the upper band (black arrow) additionally contains BSEP exon 3 (52 + 165 bp). The *ABCB11* donor splice-site mutation c.150 + 3A > C results in complete skipping of exon 3 *in vitro*. bp: base pairs, ex: exon, for: forward primer, in: intron, rev: reverse primer, WT: wildtype.

To analyse splicing outcome of c.150 + 3A > C, BSEP exon 3 and flanking intron regions with or without the nucleotide exchange (A > C) were cloned into a heterologous splicing reporter pHSR. After transient transfection into HepG2 or HEK293 cells, RNA extraction, reverse transcription and PCR were performed. Separation by gel electrophoresis (Fig. 2) resulted in a product of about 165 bp from cells transfected with the parental minigene pHSR. Insertion of BSEP exon 3 led to a shift of plus ∼50 bp indicating that splice-sites were correctly recognised. In contrast, in presence of c.150 + 3A > C the shift was missing and the PCR product had the same size as seen for cells transfected with the empty minigene (Fig. 2). Sequencing revealed that both 165 bp products consist only of the amplified first two minigene exons whereas the larger product additionally contains 52 bp of inserted BSEP exon 3. In conclusion, the minigene assay indicates that BSEP c.150 + 3A > C leads to complete exon-skipping *in vitro*. The original uncropped gel is available as Supplementary Fig. S1.

### RNA analysis in human liver tissue

For analysis of the patient’s liver tissue, formalin-fixed paraffin-embedded (FFPE) tissue of the explanted liver was used in case of child 1 whereas for child 2, a snap frozen liver biopsy was available.

**Figure 3:**
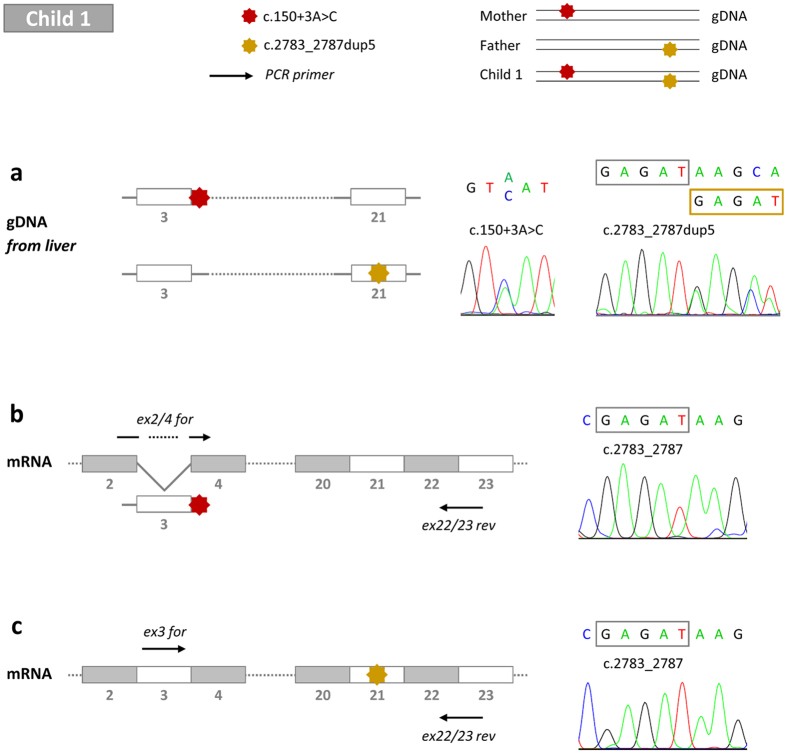
gDNA and mRNA sequencing from liver tissue of child 1. For child 1, the *ABCB11* splice-site mutation c.150 + 3A > C (red) is inherited by the mother whereas c.2783_2787dup5 (yellow) is transmitted by the father. (**a**) These mutations were detectable by sequencing of gDNA isolated from explanted liver of child 1. (**b**,**c**) For mRNA analysis, PCR forward primers *ex2/4_for* and *ex3_for* were used together with *ex22/23_rev*. Sequencing of BSEP exon 21 of both PCR products revealed the wildtype sequence. The GAGAT duplication (c.2783_2787dup5) was not detectable in mRNA transcripts with or without exon 3. ex: exon, for: forward primer, gDNA: genomic DNA, mRNA: messenger RNA, rev: reverse primer.

gDNA and RNA were isolated from liver tissue of child 1. The splice-site mutation as well as the duplication, located on different alleles, was verified on gDNA level (Fig. 3a). However, c.2783_2787dup5 was not detectable on mRNA level (Fig. 3b,c) strongly suggesting that mRNA transcripts from the affiliated allele are completely degraded. The duplication results in a premature termination codon (PTC) in exon 22, therefore, nonsense-mediated mRNA decay (NMD) is the most likely mechanism of mRNA degradation^20^,^21^,^22^. As a consequence, all proven BSEP mRNA transcripts must arise from the allele carrying the splice-site mutation, either excluding (Fig. 3b) or including (Fig. 3c) exon 3. The use of specific PCR forward primers made it possible to distinguish mRNA transcripts with or without exon 3.

**Figure 4:**
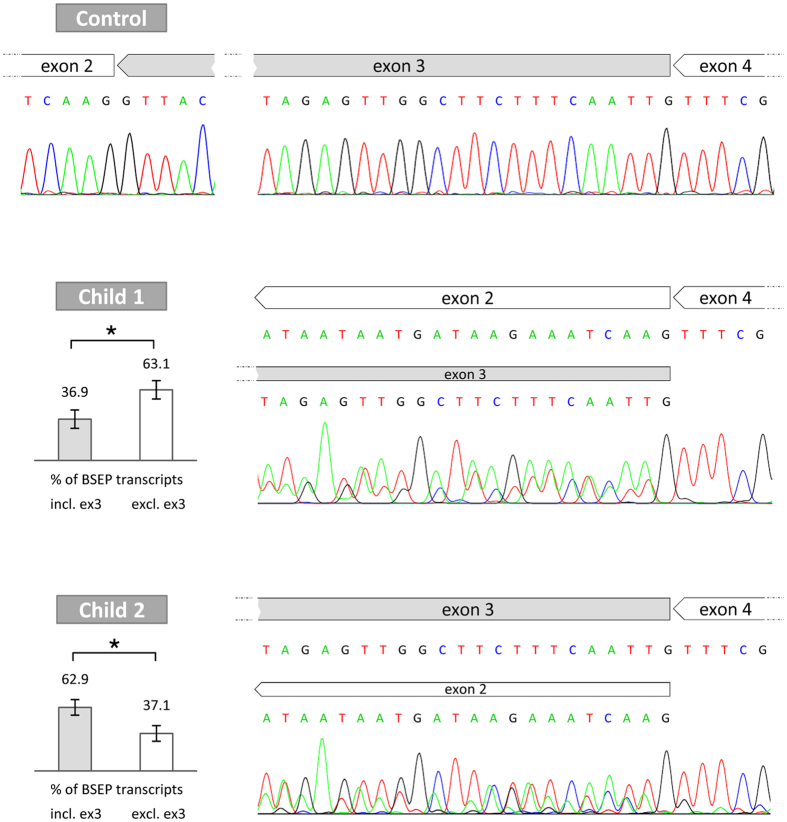
BSEP mRNA splicing effect due to c.150 + 3A > C in liver tissues of child 1 and 2. RNA from human liver tissue was used for reverse transcription and subsequent PCR. Reverse sequencing results are depicted in reverse complement. PCR product sequencing of control liver tissue displays clear peaks of BSEP exons 2, 3 and 4 (n = 14). In contrast, PCR product sequencing of child 1 and 2 shows overlapping peaks of BSEP exons 2 and 3, proving co-existence of mRNA transcripts with and without BSEP exon 3. Peak areas of each nucleotide within the overlap were measured and revealed amounts of 36.9 ± 8.2% for transcripts including exon 3 whereas in 63.1 ± 8.2% of transcripts exon 3 is missing in case of child 1. For child 2, higher amounts of transcripts with exon 3 (62.9 ± 7.7%) compared to transcripts without exon 3 (37.1 ± 7.7%) were calculated. Percentages are given as mean values with standard deviations, *significantly different with p < 0.0001 proved by the student’s t-test. ex: exon, excl.: excluding, incl.: including.

In order to quantify the amounts of mRNA transcripts including or excluding exon 3, the relevant area was sequenced for the patients’ liver tissues and 14 normal human liver samples. Reverse sequencing starting from exon 4 towards exon 1 (displayed as reverse complement sequence; Fig. 4) is depicted to avoid an additional overlap due to an insertion in isoform BSEP-B^23^. All control samples showed clear signals of exon 4, 3, and 2. In contrast, BSEP mRNA of child 1 and child 2 revealed an overlapping sequence composed of exon 2 and 3. The relative distribution of mRNA transcripts including and excluding exon 3 was calculated. Peak areas for each nucleotide of the overlap were matched and revealed for child 1 that skipping due to c.150 + 3A > C was observed in 63.1 ± 8.2% of transcripts (upper white bar, Fig. 4). Providing that detectable BSEP mRNA of child 1 entirely originates from the allele containing the splice-site mutation, it can be concluded that exon 3 is only partially skipped in the presence of c.150 + 3A > C in the patient’s liver tissue. Furthermore, liver-specific mRNA sequencing of child 2 revealed that exon 3 is skipped in 37.1 ± 7.7% of transcripts (lower white bar, Fig. 4). In contrast to child 1, mRNA transcripts of both alleles of child 2 were included. The second mutation c.2494C > T (p.R832C) of child 2 does not affect mRNA processing.

**Figure 5:**
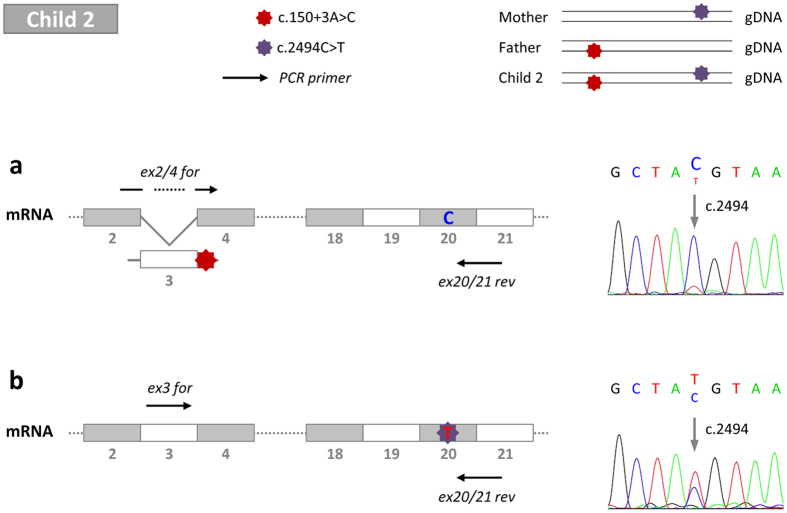
BSEP mRNA analysis of splicing extent due to c.150 + 3A > C in liver of child 2. The splice-site mutation c.150 + 3A > C on the paternal allele is shown in red. The missense mutation c.2494C > T (p.R832C) inherited by the mother is displayed in purple. RNA from the liver biopsy was used for reverse transcription. (**a**,**b**) PCR forward primers *ex2/4_for* and *ex3_for* were combined with *ex20/21_rev*. Exon 20 was sequenced from both PCR products. Appearance of cytosine (C) or thymine (T) at position 2494 indicates the extent of exon-skipping caused by c.150 + 3A > C. (**a**) When primer *ex2/4_for* was used and hence exon 3 was absent, the main sequencing peak at c.2494 results from C. (**b**) In contrast, presence of exon 3 is associated with T to larger and C to lower amounts at that position. Occurrence of both alleles using *ex3_for* confirms that c.150 + 3A > C causes only partial exon-skipping in the patients’ liver tissues. for: forward primer, gDNA: genomic DNA, mRNA: messenger RNA, rev: reverse primer.

Based on the missense mutation p.R832C, it was possible to distinguish the patient’s alleles and to further prove exon-skipping in another approach (Fig. 5). Two different specific forward primers were combined with a reverse primer covering position c.2494 of the nucleotide exchange C > T in exon 20. Primer *ex2/4* is complementary to the exon transition of exon 2 and 4 in case of skipped exon 3 whereas primer *ex3* only binds when this exon is present. In the setting with a skipped exon 3, cytosine (C) from the wildtype sequence was almost exclusively found in exon 20 at position c.2494, whereas thymine (T) appears only to a very small amount (Fig. 5a). The ratio of peak areas (C/T) was 22/1, indicating that exon-skipping predominantly affects the allele containing the splice-site mutation. On the contrary, when exon 3 was present, the T peak was higher at c.2494 than the C peak (Fig. 5b) with a ratio of 2/1 (T/C). In summary, correctly spliced mRNA transcripts of child 2 arose not only from the maternal allele carrying c.2494C > T but also to some extent from the paternal allele despite the splice-site mutation (Fig. 4, lower panel, Fig. 5b).

### BSEP protein expression

**Figure 6:**
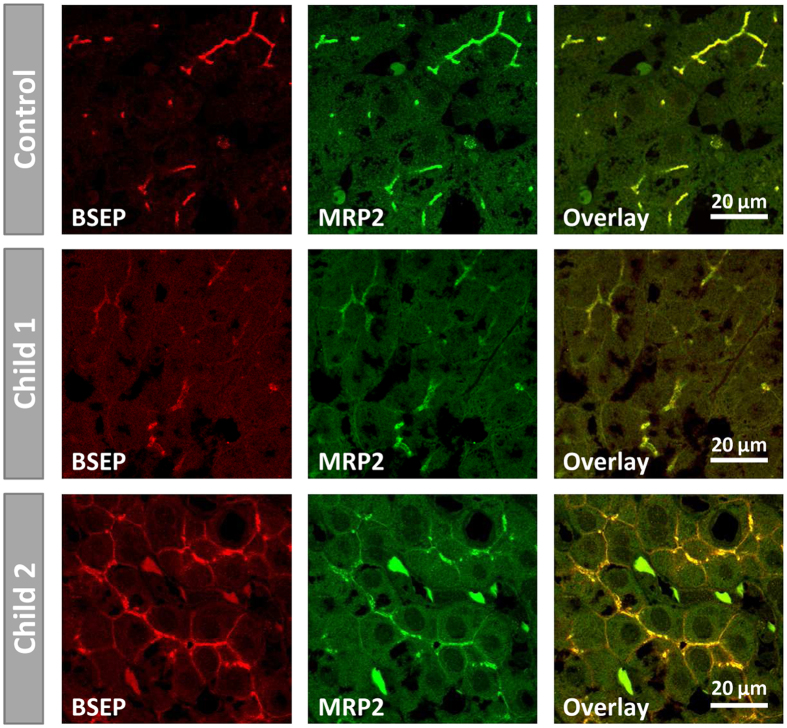
Detection of BSEP and MRP2 in human liver by immunofluorescence. BSEP and MRP2 are clearly stainable at the canalicular membrane of hepatocytes in normal human liver tissue (control). Both proteins are also detectable in liver tissue of child 1 and 2. For child 1, BSEP and MRP2 expression in the explanted liver is detectable to a lesser extent and slightly inhomogeneous as compared to control tissue. For child 2, both proteins show a distinct staining pattern. Additionally, a weak signal for BSEP and MRP2 at the basolateral membrane was observed in hepatocytes of child 2.

In normal human liver tissue BSEP and the bilirubin transporter MRP2 co-localise within the canalicular membrane and show a distinct immunoreactivity. Although to a lesser extent, BSEP was detectable in livers of both children co-localising with MRP2 (Fig. 6). In child 1, c.2783_2787dup5 excludes protein expression because BSEP mRNA transcripts from the affiliated allele were not observable (Fig. 3b,c). Therefore, detection of canalicular BSEP expression in child 1 confirms that mRNA splicing and processing from the allele with the splice-site mutation works properly to some extent.

## Discussion

Progressive familial intrahepatic cholestasis (PFIC) represents a group of genetically diverse cholestatic liver diseases due to defects of distinct transporter proteins. Three different genes associated with PFIC are *ATP8B1* (FIC1), *ABCB11* (BSEP) and *ABCB4* (MDR3)^24^,^25^,^26^, leading to PFIC type 1, 2 or 3, respectively. Recently, another PFIC-contributing gene was identified by Sambrotta and colleagues^27^. Mutations in the tight junction protein 2 gene (*TJP2*) can lead to PFIC type 4. The severity and possible treatment options of PFIC phenotypes are determined by the molecular consequences of particular mutations, and may, for example, depend on residual targeting and transport activity^28^,^29^,^30^. Hence, a detailed knowledge of the molecular effects of causative mutations is desirable for optimal disease management of individual PFIC patients. In the past, ursodeoxycholic acid (UDCA) has been shown to be effective in treatment of some PFIC-2 patients^31^,^32^,^33^,^34^. More specifically, UDCA increases BSEP activity of the BRIC-2-associated mutation p.A570T *in vitro*^35^. Furthermore, expression of BSEP with the common mutations p.E297G and p.D482G at the cell surface could be increased by the chaperone 4-phenylbutyrate^30^,^36^. Further studies showed improved liver function using 4-phenylbutyrate in patients^37^,^38^,^39^. The majority of known BSEP mutations are missense mutations due to nucleotide exchanges within exons^9^,^12^, which may affect pre-mRNA splicing^40^ or result in a non-functional, misfolded, mistargeted or instable protein with increased turnover^30^. Furthermore, several nonsense and frameshift mutations of BSEP are known. These mutations implicate PTCs, which may entail NMD, a mechanism for mRNA quality control^20^,^21^,^22^. During mRNA maturation, introns are excised from pre-mRNA and exon/exon borders are tagged by exon junction complexes (EJCs). These complexes are normally removed by the ribosome during the first translation cycle. A PTC stops the ribosome so that downstream EJCs persist. A remaining EJC triggers the recruitment of a termination complex of surveillance factors, which finally initiate mRNA degradation by exoribonucleases^20^. A prerequisite for NMD to occur is the presence of at least one retained EJC downstream to the PTC^41^. This is fulfilled in child 1 with BSEP c.2783_2787dup5. The PTC arises in exon 22 (c.3022–3024) upstream of five exon/exon borders with remaining EJCs. The duplication of GAGAT is detectable on gDNA but not on mRNA level from the same liver sample of child 1 (Fig. 3). Thus, it can be assumed that the corresponding mRNA transcripts are effectively removed by NMD. Moreover, absent BSEP protein expression is reported for a patient homozygous for c.2783_2787dup5^42^. Child 1 has been described earlier by Noe *et al*.^43^, and the mutations were referred as intron 4(+3)A > C for c.150 + 3A > C and K930X for p.K930Efs79X. Interestingly, in a liver biopsy taken from the patient at the age of 20 months BSEP was not detectable by immunohistochemical staining^43^. Our immunofluorescent staining of the explanted liver obtained at the age of 3 years showed a detectable but reduced canalicular BSEP expression (Fig. 6)^44^. The discrepancy of BSEP staining in this patient’s liver tissue may have different reasons. It is known that the expression of hepatobiliary transporters increases during development^45^ with lower expression of BSEP, MRP2, MDR3 and FIC1 during the fetal period. Therefore, immunoreactivity may have exceeded a threshold at 3 years (this study) as compared to 20 months^43^. In both studies, BSEP antibodies directed against the same C-terminal epitope were used^46^, however, they were raised in two unrelated rabbits resulting in different affinities. Lastly, the sensitivity of immunohistochemistry (Noe *et al*.^43^) as compared to immunofluorescence (this study) differs to some extent and may also influence the detection limit. Finally, canalicular BSEP protein immunoreactivity in child 1 is in line with the detection of correct BSEP mRNA transcripts containing exon 3 but not the duplication. Exon 3 consists of 52 bp, therefore skipping of this exon results in a frameshift with the first PTC at codon position 44. Nevertheless, mRNA transcripts with skipped exon 3 were detectable (Figs 3 and 4). Not only mRNAs with a PTC in the last exon are insensible to NMD because of a lacking downstream EJC, but also mRNAs containing a PTC close to the translation initiation site may escape NMD, when an alternative start codon is present^47^,^48^. Indeed, an alternative in-frame start codon is found at codon 62 of BSEP wildtype cDNA. Initiation of translation at this position would allow the ribosome to translate the sequence between exon 4 and 27 (resulting in a protein with 1260 amino acids), removing all EJCs and thereby protecting BSEP mRNA from NMD. Splice-site variants also have an impact on mRNA processing. About 6–7% of more than 380 known *ABCB11* variants (affecting exons or the adjacent 15 intronic nucleotides) are intronic splice-site variants (Table 1). Most of them concern the terminal intronic nucleotides (+1/+2 or −1/−2). These core or obligate nucleotides are essential for splice-site recognition^15^,^49^ explaining the detrimental effects of these *ABCB11* splice-site mutations. Within this work, we present a detailed characterisation of the more distally located donor splice-site mutation c.150 + 3A > C associated with a PFIC-2 phenotype making use of RNA analysis obtained from the patients’ liver samples. We found this variant in two out of 140 samples of children with a suspected BSEP deficiency analysed in our laboratory. In this highly selected cohort, the allele frequency is 0.7% (2/2 × 140). Otherwise, its allele frequency is most likely <0.2%, because there is no positive hit for this variant in more than 6000 sequences which are available on Exome Variant Server, NHLBI GO Exome Sequencing Project (ESP), Seattle, WA (URL: http://evs.gs.washington.edu/EVS/) [February 2016 accessed]. While *in silico* analyses as well as the minigene assay suggests a complete exon-skipping, mRNA data from the patients’ liver tissues clearly demonstrate that exon-skipping is only partial in the presence of c.150 + 3A > C. Therefore, results obtained from minigene constructs as used in our study, by Byrne *et al*.^40^ or by van der Woerd *et al*.^50^ should be interpreted carefully because they do not fully represent the situation in human tissue. Taking into account that in child 1 BSEP mRNA transcripts from the paternal allele are completely removed by NMD, BSEP mRNA entirely originates from the maternal allele with c.150 + 3A > C. Analysis of the overlapping sequences of exon 2 and 3 revealed an amount of 63% transcripts lacking exon 3 (Fig. 4), in other words, one third of available BSEP mRNA is correctly spliced despite c.150 + 3A > C. While Byrne *et al*. demonstrated the impact of exonic variants of *ABCB11* on mRNA processing^40^, our data show that interference of splice-site variants with mRNA abundance may be variable. Our finding of partial exon-skipping is important in view of potential therapies, since modulation of splice processes represents a therapeutic approach for some genetic diseases. Analyses with modified U1 small nuclear RNA adapted to mutated donor splice-site sequences of *ATP8B1* were recently described to increase the amount of correctly spliced products *in vitro*^50^. Such approaches require detailed knowledge of the effects of individual mutations on splicing^51^,^52^. For example, glucocorticoids have been described to interfere with transcription and mRNA processing on different levels, including alternative promoter usage^53^, induction of shortened protein variants^54^ or inhibition of pre-mRNA splicing^55^. Interestingly, child 2 of our study was successfully treated by glucocorticoids^16^. His second mutation p.R832C is clearly associated to PFIC-2^9^. Although it is under suspicion to cause aberrant splicing^40^, normal protein expression of BSEP^R832C^ was observed^9^, and loss of function is the likely reason for its severity. Although the beneficial effect of glucocorticoids may be due to a recovered transport activity of BSEP^R832C^, it must be considered that glucocorticoids improve splicing efficiency of BSEP^150+3A>C^. In conclusion, different mechanisms of defective BSEP mRNA processing cause BSEP deficiency; their recognition provide individual therapy targets.

## Patients, Materials and Methods

### Patients

The study was performed according to the guidelines of the Declaration of Helsinki and written informed consent from each patient (or parents) was obtained. Research on hepatobiliary transporters in human tissue or blood samples as presented in this study is approved by the ethical review committee of the Medical Faculty of the Heinrich Heine University Düsseldorf (approval number 2875).

### Genomic DNA sequencing

Whole blood was used for genomic DNA (gDNA) extraction using MagNA Pure LC 2.0 DNA Isolation Kit I (Roche, Mannheim, Germany). gDNA from formalin-fixed paraffin-embedded (FFPE) liver tissue was isolated using EZ1 DNA Tissue Kit (Qiagen, Hilden, Germany) after pre-treatment of 10 μm slices with xylene and ethanol. *ABCB11* coding exons with flanking intron regions were amplified by polymerase chain reaction (PCR; primer: Supplementary Table S2), and sequenced^6^,^7^. Reference sequence was NM_003742.2 (Gene ID: 8647, Entrez Gene). Genetic variants were termed according to the rules of the Human Genome Variation Society^56^. First *ABCB11* coding exon is counted as exon 1, adenine of ATG is denoted as c.1, IVS4 indicates intervening sequence among coding exons 3 and 4.

### Minigene construct preparation

gDNA was isolated from whole blood of a healthy person. *ABCB11* exon 3 and additional intronic 800 bp 5′ and 500 bp 3′ were amplified by PCR using *Taq* Polymerase (Qiagen). TOPO TA cloning and transformation into TOP10 *Escherichia coli* (Invitrogen, Carlsbad, CA, USA) were performed. Plasmid DNA was isolated from appropriate clones using HiSpeed Plasmid Purification Maxi Kit (Qiagen). The human immunodeficiency virus (HIV)1-based long terminal repeat heterologous splicing reporter (pHSR) has been described in Betz *et al*.^18^. Purified TOPO plasmid containing BSEP exon 3 and minigene pHSR were cut with *Eco*RI (NEB, Ipswich, MA, USA). Linearised dephosphorylated minigene (∼4.5 kB) and BSEP exon 3 with intronic sequences (∼1.3 kB) were ligated using T4 DNA Ligase (Promega, Mannheim, Germany). An eligible clone of minigene containing BSEP exon 3 with a wildtype (WT) donor splice-site (pHSR-BSEP_ex3_WT) was extended and used for mutagenesis (QuikChange Multi Site-Directed Mutagenesis Kit, Stratagene, La Jolla, CA, USA). Final constructs pHSR-BSEP_ex3_WT and pHSR-BSEP_ex3_c.150 + 3A > C were verified by sequencing.

### Cell culture and transfection

Hepatocellular carcinoma (Hep)G2 cells^57^ were cultivated in DMEM Ham’s F12 (Biochrom, Berlin, Germany) supplemented with 10% (v/v) FCS (PAA, Coelbe, Germany) at 37 °C in 5% CO_2_. Cells were transfected 24 h after seeding in 6-well plates with 1 μg DNA of pHSR, pHSR-BSEP_ex3_WT or pHSR-BSEP_ex3_c.150 + 3A > C, using X-tremeGENE HP DNA transfection reagent (Roche).

### Splicing analysis using a minigene assay

48 h after transient transfection, total RNA was extracted from cells via the Maxwell16 system (LEV simplyRNA Tissue Kit, Promega). RNA was transcribed into complementary DNA (cDNA) using QuantiTect Reverse Transcription Kit (Qiagen). 3 μl of each cDNA were amplified by Phusion Polymerase (Thermo Fisher, Waltham, MA, USA) with primers surrounding the exon of interest insertion site (Supplementary Table S2). PCR products were analysed by agarose gel electrophoresis and proven by sequencing of extracted DNA. Gel was containing 1.5% agarose in Tris-acetate-EDTA buffer. Smart Ladder (Eurogentec, Kaneka Corporation, Osaka, Japan) was used as a marker.

### Splicing analysis in human liver tissue

Liver tissue of both patients and controls was used for splicing analysis. For child 1, RNA was extracted from FFPE explanted liver tissue using LEV Blood DNA Kit with additional buffers (Promega). Two 10 μm slices were incubated in 200 μl RNA incubation buffer at 80 °C for 10 min and afterwards at 56 °C for 45 min. After adding 500 μl RNA lysis buffer, samples were processed in the Maxwell16 system (Promega). RNA was isolated from a snap frozen liver biopsy of child 2 and snap frozen normal liver specimens of 14 patients who underwent surgery because of liver metastasis. cDNA synthesis was performed with GoScript Reverse Transcription System (Promega) at 50 °C using primers *ex0_for* and *ex23/24_rev* (Supplementary Table S2). Subsequent PCR reactions were conducted differing in their forward primers: (1) *ex0/1_for*, (2) *ex2/4_for*, and (3) *ex3_for*, each in combination with reverse primer *ex22/23_rev* for child 1 or *ex20/21_rev* for child 2 and controls. Samples of PCR (1) were sequenced with *ex0/1_for* and *ex4/5_rev*. BSEP mRNA exons 1 to 4 were analysed by reverse sequencing because the readout of forward sequencing was limited by an alternative coding exon 2 of isoform BSEP-B^23^. In PCR (2) *ex2/4_for* spans the junction of exon 2 and 4 and attaches to the template only when exon 3 is skipped. In (3) *ex3_for* just binds in the presence of exon 3. PCR products of (2) and (3) were sequenced with *ex18_for* and *ex22/23_rev* (child 1) or *ex20/21_rev* (child 2) to carry out the allelic assignment by the second mutation.

### Immunofluorescence of liver tissue

Immunofluorescence was performed according to standard protocols^58^,^59^ using Advalytix Slide Booster (Implen, Munich, Germany) for antibody incubation. A polyclonal rabbit antibody was raised against 13 C-terminal amino acids of BSEP compliant with Noe *et al*.^46^. A mix of monoclonal mouse antibodies for multidrug resistance-associated protein 2 (MRP2; M2I-4 and M2III-6, 1:25, Enzo Life Sciences, Lörrach, Germany)^59^ and BSEP antibody (K24, 1:25) were applied for 2 h at 29 °C. Tissue samples were incubated with secondary antibodies conjugated to Alexa Flour 488 (goat anti mouse, A11029, green) or 546 (goat anti rabbit, A11035, red) (1:250, Invitrogen) for 1 h at 27 °C. Specimens were analysed with a LSM510 confocal laser scanning microscope (Zeiss, Jena, Germany).

### Statistical analysis

To quantify the ratio of exon 3 inclusion and exclusion, sequencing traces for each base were separated by CodonCode Aligner (V.4.2.5, CodonCode Corporation, Dedham, MA, USA). The area underneath each peak within the overlap was measured using ImageJ^60^ and calculated as a fraction of the combined signal at each position. Values were assigned to exon 2 or 3 and averaged. For detailed proceeding see Supplementary Fig. S2. Paired student’s t-test was used with p < 0.0001 considered as significantly different.

## Additional Information

**How to cite this article**: Dröge, C. *et al*. Exon-skipping and mRNA decay in human liver tissue: molecular consequences of pathogenic bile salt export pump mutations. *Sci. Rep.* **6**, 24827; doi: 10.1038/srep24827 (2016).

## Supplementary Material

Supplementary Information

## Figures and Tables

**Table 1:** Clinical relevant *ABCB11* mutations affecting intronic splice-site consensus sequences.

*ABCB11* mutations affecting	donor splice-site		acceptor splice-site	
obligate dinucleotides	c.611 + 1G > A	c.2178 + 1G > T	c.77-1G > C	c.2179-2A > G
	c.908 + 1delG	c.2343 + 1G > T	c.99-1G > T	c.2611-2A > T
	c.908 + 1G > A	c.2343 + 2T > C	c.390-1G > A	c.3057-2A > G
	c.908 + 1G > T	c.3213 + 1delG	c.1639-1A > G*	
	c.2178 + 1G > A		c.2012-2A > G*	
distal positions	c.76 + 3G > T	c.3213 + 4A > G	c.77-7C > A^#^	c.2012-8T > G
	c.150 + 3A > C	c.3213 + 5G > A	c.1435-13_-8del	

For *ABCB11*, 17 intronic mutations concerning the obligate dinucleotides (+1/+2 and −1/−2) are known. Additionally, seven intronic variants located in vicinity to exon/intron borders (+/−10) are described. New mutations recently identified in patients analysed in Düsseldorf are marked with an asterisk (*), others are referenced in^12^, ^#^ is a variant of child 1.
