# Supplementary material for: Exon-skipping and mRNA decay in human liver tissue: molecular consequences of pathogenic bile salt export pump mutations
Source: Sci Rep. 2016 Apr 26;6:24827. doi: 10.1038/srep24827 (PMC4845019; doi:10.1038/srep24827)
Supplement: Supplementary Information [file srep24827-s1.pdf]

Supplementary Information for:

Exon-skipping and mRNA decay in human liver tissue: molecular consequences of pathogenic bile salt export pump mutations

Carola Dröge[1], Heiner Schaal[2], Guido Engelmann[3], Daniel Wenning[4], Dieter Häussinger[1], Ralf Kubitz[1,5*]

[1]Department of Gastroenterology, Hepatology and Infectious Diseases, University Hospital, Heinrich Heine University, Düsseldorf, Germany

[2]Institute of Virology, Heinrich Heine University, Düsseldorf, Germany

[3]Department of Pediatrics, Lukashospital, Neuss, Germany

[4]Department of General Pediatrics, University Hospital, Heidelberg, Germany

[5]Medical Clinic I, Bethanien Hospital, Moers, Germany

[*]Corresponding author

Supplementary Fig. S1

Splicing analysis of c.150+3A>C by a minigene assay (Original uncropped gel).

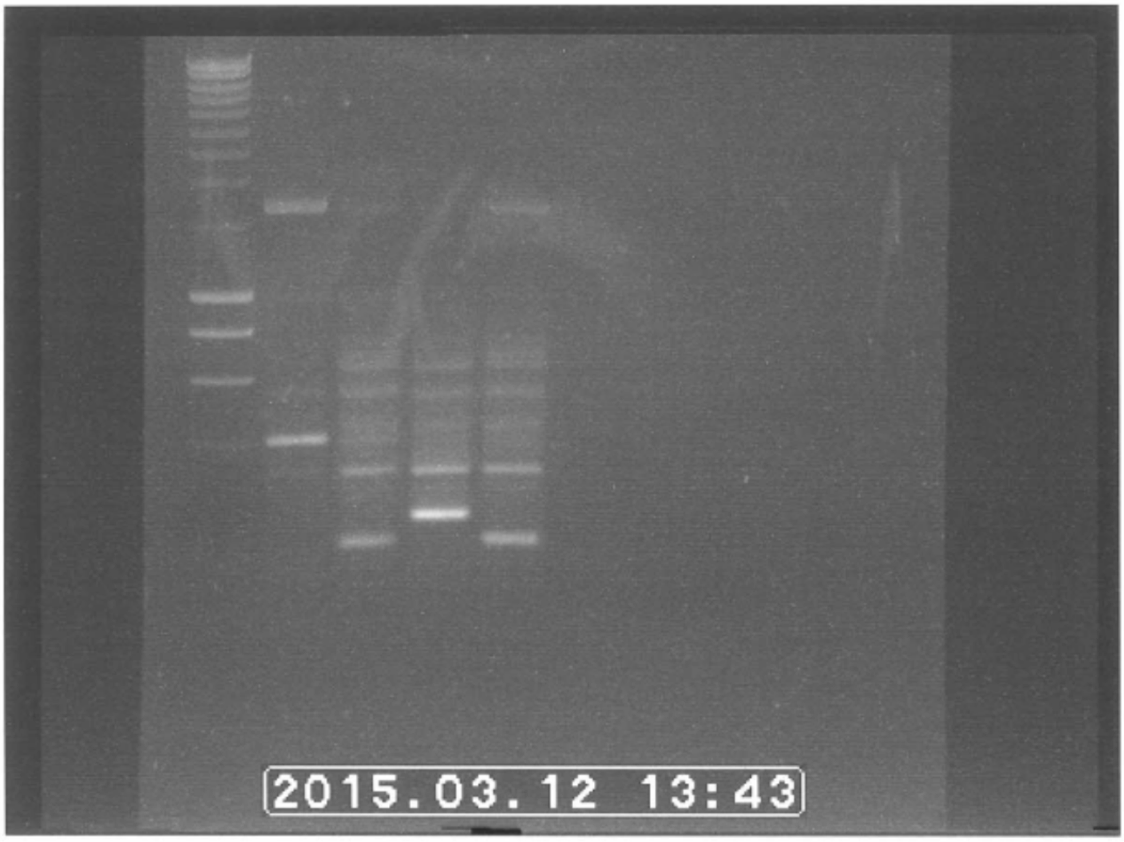

Supplementary Fig. S2

Determining relative amounts of mRNA transcripts with or without exon 3.

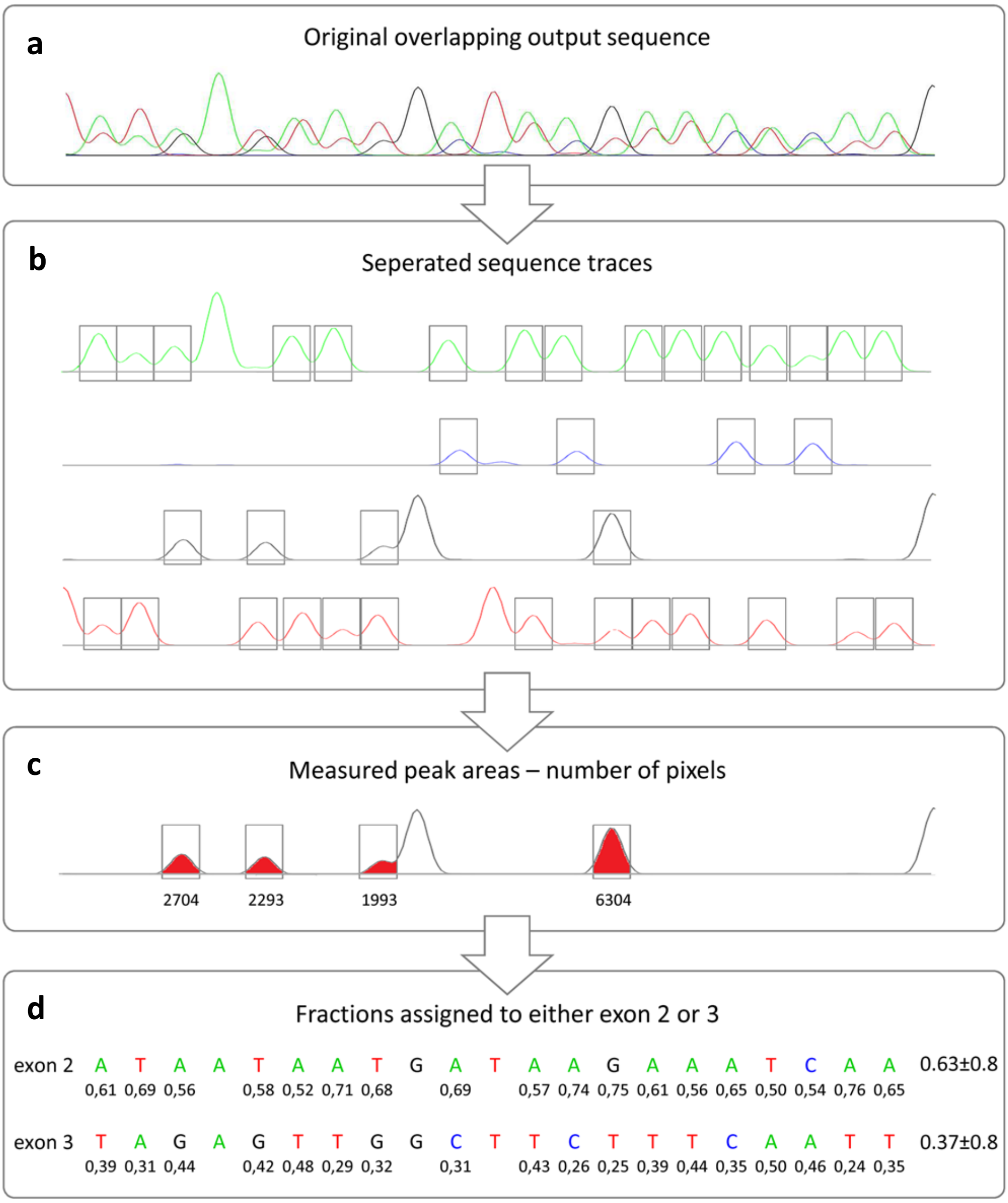

The method is elucidated based on the overlapping sequence from mRNA analysis of child 1. **a** Original sequencing output consisted of overlapping peaks. **b** Sequencing traces were separated for each base using CodonCode Aligner. Single peaks were encircled with boxes of the same width. **c** Areas underneath each peak (marked in red) were quantified by ImageJ, given as the number of pixel. **d** Values of area measurements were calculated as a fraction of the combined signal at each nucleotide position and assigned to exon 2 or 3. Mean values and standard deviation for signals related to exon 2 or 3 were calculated.

Supplementary Table S1

ABCB11 variants detected in two PFIC-2 patients.

**Child 1**					
**exonic variants**	**nucleotide change (c.)**	**protein effect (p.)**	**reference SNP cluster ID**	**zygosity**	**shown RNA effect**
coding ex 12	1331C>T	V444A	rs2287622	heterozygous	
coding ex 21	2783_2787dup5	K930Efs79X	-	heterozygous	mRNA degradation (NMD)
coding ex 23	3084A>G	A1028A	rs497692	homozygous	
**intronic variants**	**nucleotide change (c.)**		**reference SNP cluster ID**	**zygosity**	**shown RNA effect**
IVS2	77-7C>A		-	heterozygous	
IVS4	150+3A>C		rs387906354	heterozygous	exon skipping
IVS9	909-15A>G		rs2287618	heterozygous	
IVS13	1434+70C>T		rs2287623	heterozygous	
IVS14	1638+32T>C		rs2241340	heterozygous	
IVS18	2179-17C>A		rs853772	homozygous	

**Child 2**					
**exonic variants**	**nucleotide change (c.)**	**protein effect (p.)**	**reference SNP cluster ID**	**zygosity**	**shown RNA effect**
coding ex 12	1331C>T	V444A	rs2287622	heterozygous	
coding ex 20	2494C>T	R832C	-	heterozygous	
coding ex 23	3084A>G	A1028A	rs497692	heterozygous	
**intronic variants**	**nucleotide change (c.)**		**reference SNP cluster ID**	**zygosity**	**shown RNA effect**
IVS4	150+3A>C		rs387906354	heterozygous	exon skipping
IVS9	909-15A>G		rs2287618	heterozygous	
IVS13	1434+70C>T		rs2287623	heterozygous	
IVS18	2179-17C>A		rs853772	heterozygous	
IVS19	2344-17T>C		rs853789	heterozygous	

Supplementary Table S2

Primers used to characterise the relevant *ABCB11* (BSEP) mutations of two PFIC-2 patients.

**gDNA analyses from blood**	**primer: 5' → 3'**
seq_ex3_for	TCACCACCTAGGGAGAATTTC
seq_ex3_rev	AGTTATTCAGCATGTTATCAC
seq_ex20_for	CAGTAAGTGATTTAGTTATTTTTC
seq_ex20_rev	CACTGGTCCCTATTCCATAG
seq_ex21_for	TGAATTGTACACTTAAGAATG
seq_ex21_rev	GGCTGACAGCTTCCTTCAGTC
**gDNA analyses from liver**	**primer: 5' → 3'**
seq_liv_ex3_for	ATCACCACCTAGGGAGAATTTCCC
seq_liv_ex3_rev	TGCCAATATGACTAAAGATTTAACACTCCC
seq_liv_ex21_for	TGTCTGAGACGGGTTGATTGC
seq_liv_ex21_rev	TACTCGTTTTTAACAGTTTGTCTGATAGCC
**minigene assay**	**primer: 5' → 3'**
mini_ex3_for	ACTTCGAACTTTCCTAGATAGAGTAGGG
mini_ex3_rev	GTTTCGAATAATTGAGTGGCAGAGTTCG
mut_c.150+3A>C	AGAGTTGGCTTCTTTCAATTGGTCATAAAACTGTGTTGATTTATGGC
pHSR_ex1_for	CCGAACAGGGACTTGAAAGCG
pHSR_ex2_rev	TGCAGGAACAAGCAAGGCC
**mRNA analyses from liver**	**primer: 5' → 3'**
ex0_for	AACTCTCCACAGTGGAGTCC
ex23/24_rev	CCCCTGGAAGTTGTCCCATTT
ex0/1_for	TCACAGGGTCGTTGGCTGTGGGTTGC
ex2/4_for	TGATGGTTTTGAGTCAGATAAATCATATAATAATGATAAGAAATCAAGTTTCGG
ex3_for	AGATGAGAAGAAAGGTGATGGCGTTAGAGTTGGC
ex22/23_rev	AACTGCAGAGATCACCCTGAACACATAGC
ex20/21_rev	ATCTGAGAGCCGGCAGCCCCTTGAACTTGG
ex4/5_rev	TCTCGATGTTCAGCAACCCAC
ex18_for	AACGGGACAGTCACACC
